# Innovation by patients with rare diseases and chronic needs

**DOI:** 10.1186/s13023-015-0257-2

**Published:** 2015-04-09

**Authors:** Pedro Oliveira, Leid Zejnilovic, Helena Canhão, Eric von Hippel

**Affiliations:** UCP - Católica-Lisbon School of Business and Economics, Lisbon, Portugal; Department of Engineering and Public Policy, Carnegie Mellon University, Pittsburgh, USA; University of Lisbon School of Medicine and Santa Maria Hospital, Lisbon, Portugal; MIT Sloan School of Management, Cambridge, MA USA

**Keywords:** Patient Innovation, User Innovation in Health, Diffusion of Innovation, Rare Diseases

## Abstract

**Abstract:**

We provide the first empirical exploration of disease-related innovation by patients and their caregivers. Our aims were to explore to what degree do patients develop innovative solutions; how many of these are unique developments; and do these solutions have positive perceived impact on the patients’ overall quality of life? In addition, we explored the factors associated with patient innovation development, and sharing of the solutions that the patients developed.

**Methods:**

We administered a questionnaire via telephone interviewing to a sample of 500 rare disease patients and caregivers. The solutions reported were pre-screened by the authors for their fit with the self-developed innovation aim of the study. All the reported solutions were then validated for their novelty by two medical professionals. Logistic regression models were used to test the relationships between our key variables, patient innovation and solution sharing.

**Results:**

263 (53%) of our survey respondents reported developing and using a solution to improve management of their diseases. An initial screening removed 81 (16%) solutions for being an obvious misfit to the self-developed innovation aim of the study. This lowered the sample of potentially innovative solutions to 182 (36%). Assessment of novelty and usefulness of the solutions, conducted by two medical evaluators, confirmed that 40 solutions (8%) were indeed novel, while the remaining 142 (28%) were already known to medicine. The likelihood of patient innovation increased as the education level increased (OR 2, p < 0.05), and as their perception of limitations imposed by their disease increased (OR 1.3, p < 0.05). 55 individuals diffused their solutions to some degree, with 50 of these sharing via direct diffusion to other patients. There is a positive relationship between the impact of a solution on the respondents’ overall quality of life and likelihood of solution sharing.

**Conclusions:**

Given that hundreds of millions of people worldwide are afflicted by rare diseases, patient and their caregivers can be a tremendous source of innovation for many who are similarly afflicted. Our findings suggest that many patients could be greatly assisted by improved diffusion of known solutions and best practices to and among patients and their caregivers.

**Electronic supplementary material:**

The online version of this article (doi:10.1186/s13023-015-0257-2) contains supplementary material, which is available to authorized users.

## Background

There are 5000 to 8000 rare diseases which, taken together, afflict up to 8% of the world’s population [1,2]. Most are genetic and chronic, and many impose significant difficulties on the daily lives of both patients and their caregivers [3]. Patients with rare diseases also tend to be underserved both clinically and scientifically [4]. Small market size, due to each diseases’ low prevalence, makes it commercially unattractive for pharmaceutical firms and other medical suppliers to invest in developing new products specifically for rare diseases [5].

High patient need coupled with low commercial activity in rare disease marketplaces creates both a need and an incentive for patients and their caregivers to innovate for themselves to help them with respect to many quality of life issues. Studies of innovation by citizens show that useful innovation by patients to serve their own needs is likely: many citizens have been found to develop and improve products for their own use in a wide range of fields, including medical care needs [6-8]. Indeed, individuals with high and unmet need for a solution often develop a solution/product for their own use before a producer introduces an improved version of the product to the market [9,10].

An increasing stream of literature argues for a more prominent role of patients and caregivers in healthcare delivery [11-14]. However, this literature does not yet consider the innovation capacity of patients and caregivers. Recent qualitative research has found that patients and caregivers are not only the drivers of institutional research [15], they invent a myriad of valuable solutions to improve their own personal medical situations. These solutions span from simple tools for everyday use, through discovery of previously unknown therapies, to highly sophisticated solutions. An example of the latter is a textile mesh support for dilated heart aorta developed by a Marfan syndrome patient [16,17]. Emergence of innovation intermediaries in healthcare [18], and initiatives to discover and share patients’ solutions (like www.patient-innovation.com) suggest that the awareness of patients’ innovation capacity is increasing. Still, there is a dearth of quantitative academic research related to patient innovation.

Patients and their caregivers who develop solutions to address some of their disease related problems can potentially give valuable contributions to the stock of knowledge about their diseases and ways to cope with them. The general value of these solutions increases if the solutions diffuse. Our objectives in this study are to explore: to what degree do patients develop innovative solutions; how many of these are unique developments; do these solutions have positive perceived impact on quality of life; and, whether the patients share their solutions and how. With this paper, we aim to provide evidence of the rare diseases patients’ innovation activity, and propose paths for further research on this important topic.

## Methods

### Data collection

We conducted a survey of patients with rare diseases with the support of an association of mental deficiencies and rare diseases located in Europe. The association has about 800 patients registered as its members and runs a help-line to support patients inquiring about administrative and disease related issues. The association documents all contacts, and together with the information on members has records of about 5000 individuals who had contacted its help-line in the period from 2009 until the end of 2012. From these 5000, the association’s staff randomly selected a sample of 1000 individuals for us to contact.

Four, trained social workers with extensive experience in working with rare diseases patients within the helpline, were appointed by the Association to administer our survey. To familiarize these individuals with the content of the survey and goals of initial testing and validation of the survey, our research team organized two training meetings, and conducted two testing/validation sessions to adjust the survey to the targeted population and clarify interpretation. The interviewers contacted individuals on the association-supplied list in sequence, continuing until we had obtained completed questionnaires from 500 subjects.

Interviewers began each telephone interview session by identifying the participating organizations and explaining the purpose and topic of the survey. They proceeded to the questionnaire itself only after receiving the subjects’ consent to participate. The interviewers made 546 calls to the patients from the help-line’s contact list, to gather 506 responses (93% response rate), of which 500 were usable for further processing. Average call duration was 32 minutes, with calls taking anywhere from 10 to 90 minutes. All the information collected by the interviewers and included in the dataset for analysis was de-identified. Patient data was analyzed as a group with no individual disclosure of classified information according to the Helsinki Declaration for human studies. Local ethics committee (Comissão de Ética do Centro Hospitalar Lisboa Norte” (Ethics Commission of the Center of Hospitals in the North Lisbon)) gave their approval for the content of the survey. The data used in this paper, from the survey and other sources, are described in Additional file 1: Table S1.

Our survey was partitioned into 7 sections. It contained 67 questions in total. All were grounded in user innovation literature. In the first section, we identified the respondents as patients or caregivers, and adapted the language of the questions that followed accordingly. We next asked about the limitations imposed by the patient’s disease upon both patients and caregivers. We then asked about perceived needs for health-related solutions, and asked whether the patient or a caregiver had developed a solution that helped them cope with the patient’s disease. Depending on the answer, respondents were branched to one of four sections; (2) no solution developed; (3) equipment or technical aid development (4) therapy development; or (5) a behavioral change developed. Respondents with developments were then asked to describe what they had developed in detail. To estimate the value provided by their solution, respondents were also asked to estimate their overall quality of life before using their solution, and after using it. We used a single-item, 7-point Likert Scale to measure the quality of life. If the respondent was a caregiver, quality of life changes for both the caregiver and the patient were asked about. The 6th section asked questions about respondent demographics, and the 7th asked about respondents’ use of the Internet and medical community memberships.

We applied a two-step procedure for assessing the novelty of the solutions. In the first step, the authors of the paper removed solutions that were an obvious misfit to the aim of the study. After the initial screening, assessment of the novelty of all the solutions developed by the respondents was conducted independently by two Ph.D. medical professionals, who each had both clinical and research expertise. These individuals had available the information about the disease and its duration, the patients’ gender, and hand-collected descriptions about each of the 251 diseases identified in the sample. In addition, two research assistants collected information about common therapeutic practices for the diseases. Each clinician-evaluator independently read through the patient solution descriptions and, based upon their expert medical knowledge, evaluated whether respondents’ novelty claims were correct, or whether the solution was already known to them or the medical practice for the disease. While evaluating they checked the medical literature and the information collected by the research assistants. The two expert evaluators had very good inter-rater agreement (kappa = 0.81). Only when both agreed that a solution was novel did we code the solution as a patient innovation. If the solution was already known to medicine, although not to the respondent describing it, the solution was coded as a redevelopment. Redevelopments are of value to individual patient developers who obtain the use of a pre-existing solution in this way, but do not add to the stock of available medical knowledge – they are not new to the world. We also asked the evaluators to assess all the solutions and judge whether in their opinion the solutions are useful or helpful for the patients. Together with the evaluators, we established three criteria to assess the solutions’ usefulness/helpfulness: (i) it helps the patients or caregivers in their daily activities; (ii) it may help in coping with the disease; and (iii) it is a cost saving alternative to something that already exists. The evaluators were asked to give one general assessment of the solutions as useful or not-useful, based on these three criteria. In addition, the evaluators indicated which of the solutions they considered to be dangerous.

### Data analysis

In our statistical analyzes, we built and tested two multivariate discrete choice models to explore what motivated respondents to innovate in the community of rare diseases patients and what drove them to share their solutions. In the first case, our dependent variable was the validated innovation. We included the following independent variables: 1) socio-demographic variables (as shown in Table 1); 2) disease prevalence, obtained from external databases (OrphaNet, Medline, NIH, etc.) and categorized into 5 categories: higher than 1/1000; 1 to 9/10,000; 1 to 9/100,000; 1 to 9 in 1,000,000; or lower than 1/1,000,000; this variable was inversely coded – the lower the prevalence, the higher is the category; it allowed us to test whether lower prevalence implies higher need for solutions and higher likelihood of patient innovation; 3) disease burden: we asked the respondents to tell us to what extent does their disease impose limitations on their life, measured on a 5-point scale, from 1 – the disease imposes no limitations on my life, to 5 – the disease imposes extreme limitations on my life; 4) medical patient community membership; it indicates whether the respondent (or the patient if appropriate) belong to any formal group/association of patients with the same disease as they do. Also, to test for non-linearity of the effects of the respondents’ age and their disease duration on innovation, we included squared terms of these two variables.

**Table 1:** **Socio-demographic characteristics of the respondents**

**Variable**	**N**	**%**	**Mean**	**SD**	**Min**	**Max**
Respondent’s Age (years)			45	13	18	84
Disease Duration (years)			12	12	0.2	65
Women	425	85				
University Degree	194	39				
Employed	255	51				
Married	322	64				
SD = standard deviation						

We built another model to test the likelihood of information disclosure. Beside the variables from the first model, we added one variable to this model: the difference in the overall quality of life between the reported values for the periods before the innovation and after the respondent used the solution; the quality of life for the two time periods was measured on a 7-point scale, from 1 – extremely low, to 7 – excellent. We conducted the analysis using two data samples: 1) full-sample with 263 reported solutions; and 2) restricted sample with 182 solutions that passed the pre-screening. The rationale for including the both samples is the following: for studying the solution sharing activity, what matters is whether the respondents believe that they have developed something; to check the robustness of the results, we included the analysis using the restricted sample.

We presented our results as raw coefficients in the tables, together with robust standard errors. Continuous variables are expressed as mean and standard deviation and discrete variables as absolute frequency. In the text, we report odds ratios and 95% confidence intervals as measures of effect. Significant p-values were set to <0.05. Stata 13 software was used for data analysis.

## Results

### Population included in our study

Respondents’ socio-demographic characteristics are described in Table 1.

As can be seen, the majority of the 500 respondents in our sample were caregivers (59%), and 85% of the respondents were women. Women also constituted 65% of all the patients in our sample.

The average age of patients in our sample was 33 years, with a standard deviation of 22 years, while the average age of the respondents was 45 years with a standard deviation of 13 years. 65% of the respondents were between 18 and 45 years old. The disease duration was 12 years with a standard deviation of 12 years. One-third of the respondents held university degrees, 51% were employed, and 64% were married or in de facto unions.

### The extent and types of patient-developed innovations

263 (53%) of our 500 respondents reported having developed a solution they regarded as novel to assist them in managing their disease. The initial screening removed 81 solutions, leaving 182 (36% of 500) potentially novel solutions. Further evaluation by the expert medical evaluators suggested that the solutions of 8% of our respondents were also evaluated as novel – “new to the world” – by our expert medical evaluators. The remaining claimed innovations that passed the initial filtering were judged by our expert evaluators to be redevelopments – novel to the patient developer, but already known to medicine. In the following tables we use the restricted sample of 182 reported as novel solutions that passed the initial screening, unless otherwise indicated.

Almost all the reported solutions were also judged by the experts to be relatively safe: out of 182, only 4 (2%) of the patients’ developments were judged to be potentially detrimental to patients’ health by the evaluators. 73 (40%) of the patients’ developments were judged to be useful, regardless of their novelty. As can be seen in Table 2, we divided patient developments into two categories, products and services. Developments were coded as products if there was a description of a medical equipment or an assistive product, as defined in Article 2.3. of ISO 9999:2011 [19]: “any product (including devices, equipment, instruments and software), especially produced or generally available, used by or for persons with disability for participation; to protect, support, train, measure or substitute for body functions/structures and activities; or to prevent impairments, activity limitations or participation restrictions.” Developments were defined as services in other cases, where we found a description of an activity or a plan of activities related to treatments or changes in strategies or behavior related to the disease. 90% of the reported solutions were categorized as services – very few were product innovations.

**Table 2:** **Type, novelty, and usefulness of reported solutions**

	**Novelty**			**Usefulness/Helpfulness**		
	**Novel to the patient (% of 142)**	**Novel to the world (% of 40)**	**Total (% of 182)**	**Dangerous (% of 4)**	**Not useful/helpful (% of 105)**	**Useful (% of 73)**
Products	4 (3%)	15 (37%)	19 (10%)	0	4 (4%)	15 (21%)
Services	138 (97%)	25 (63%)	163 (90%)	4 (100%)	101 (96%)	58 (79%)
Total (% of 182)	142 (78%)	40 (22%)	182 (100%)	4 (2%)	105 (58%)	73 (40%)

Note: Novelty and usefulness are judged by two medical professionals, kappa = 0.8.

### Examples of the solutions

Most of the solutions in our sample are technically very simple, but nonetheless offer great value to patients. Consider the case of a mother who takes care of her son, an Angelman syndrome patient. Angelman syndrome involves ataxia, inability to walk, move or balance well. The mother experimented with many strategies, recommended by the doctors, therapists, or found elsewhere, but obtained little gain for her child. By chance, at a neighbor’s child’s birthday party, she noticed her son excitedly jumping for strings to catch a floating helium-filled balloon. This gave her an idea and she experimented at home by filling a room with floating balloons. She found her child began jumping and reaching for the balloons for extended periods of time, amused by the challenge. The mother also added bands to support the knees and keep the child in an upright position. The result was significant improvement in her child’s physical abilities. Other parents to whom she described the solution also tried the balloons strategy and had positive results. This was valued as a novel solution by the medical evaluators.

Consider now a mother of a kid with cerebral palsy. This disease, among other manifestations, makes the kid to hyper salivate. Besides being a discomfort and a potential source of disease, the hyper salivation is also a cause for social exclusion. The medical solution for this problem - removing the salivary glands - is considered by this mother as too drastic and with very limited benefits. Instead, she tried solving the problem by considering strategies that allow her son socializing more easily with other people. She developed “The Cute Turtle Collar”, a kind of a turtle collar piece made of a material that absorbs well the slobber, and is both stylish and suitable to use in all weather conditions.

Several of the reported solutions included descriptions of intensive physical activity, which, according to the respondents, led to moderate improvements in their overall quality of life. Daily practice of martial arts, Muay Thai and kick-boxing up to 5 hours a day, is an uncommon choice for a Trombocitopenic purpura patient. The patient opted for this sort of exercising contrary to the advice of health professionals not to engage in physical activities that could hurt the body. The respondent reported significant decrease in frequency of hemorrhages, which she associates with the intensive practice of martial arts.

Another case from our sample, that also involves intense physical activity, is of a boy afflicted by Charcot-Marie-Tooth disease. This disease makes the patients progressively lose muscle tissue and touch sensation, and often the first visible symptoms of the disease appear in legs and hands. The boy’s daily practice of playing piano has slowed down the disease’s development in hands and masked some of the symptoms, which made the diagnostic process harder. He continued playing the piano, and the hands remained fully functional, with little or no sign of the disease, while he may need a foot surgery to maintain the desired level of functionality. Benefits of physical exercise are well-known, but, in this case, the intensity of the exercise may have changed the development of the disease. This information may be useful for medical professionals in establishing a correct diagnosis for similar cases earlier, and also as a strategy to be considered by other patients and caregivers.

The vast majority of the innovations in our sample have been developed to increase the patients’ autonomy. For example, a patient with Myasthenia gravis, an autoimmune neuromuscular junction disorder, reported designing several products custom-built according to her specifications. She described a design of one of the tools – a metal two-hook button aid that helps her button pants without assistance of others. Although an online search quickly reveals variety of button hooks, patients, as is the case described, often experiment with the design to make the one that better fit their specific condition and needs. Other solutions reported involved experimentation with the design of elements commonly found in any household; for example, optimization of the height and width of stairs to improve mobility, or design of tables and chairs with added features to increase safety of hyperactive children with cognitive limitations.

### The impact of the solutions

The majority of the respondents who reported a solution described substantial improvements in their overall quality of life as a result of using their solutions. For solutions that were new to the patient but not new to the world, the mean improvement in quality of life of the patients was 1.6 on a seven-point Likert scale. For solutions that were new to the world, the improvement in quality of life of the patients was 2 points on a seven-point Likert scale. Caregivers reported mean improvements in the quality of their lives of 1.4 for non-novel and 1.9 for novel solutions (Table 3).

**Table 3:** **Perceived difference in overall quality of life after using the solutions**

		**New to the respondent**		**New to the world**	
		**Quality of life difference**		**Quality of life difference**	
		**Patients**	**Caregivers**	**Patients**	**Caregivers**
Products	Nr. of cases	4	1	15	9
	Mean (SD)	2.2 (2.2)	0 (0)	2.2 (1.5)	1.7 (1.4)
Services	Nr. of cases	132	62	24	17
	Mean (SD)	1.6 (1.5)	1.5 (1.5)	1.8 (1.4)	2 (1.7)
Total	Nr. of cases	136	63	39	26
	Mean (SD)	1.6 (1.5)	1.4 (1.5)	2 (1.4)	1.9 (1.6)

Quality of life improvements resulting from using the innovations were significant (p < 0.001, two-tailed *t*-test). The difference in quality of life improvements was not significantly different (p < 0.21, two-tailed *t*-test) in the case of innovations that were new to the individual patient/respondent compared to new to the world.

We matched the perceptions of the solutions’ usefulness, as seen by medical experts, with the patients’ perceptions of their overall quality of life improvements due to use of their solutions. The experts judged as not helpful 68 (59%) of 115 solutions that the patients reported as beneficial for their quality of life. Also, the experts judged as useful 23 (44%) of 52 solutions for which patients reported no improvements (Table 4).

**Table 4:** **Perceptions of the solutions’ usefulness by patients and medical professionals**

		**Reported (by respondents) improvements in overall quality of life (QoL)**		
		**No change in QoL**	**QoL improved**	**Total (% of 167)**
Medical professionals’ assessments	Dangerous	1	2	3 (2%)
	Not helpful/useful	28	68	96 (57%)
	Helpful/Useful	23	45	68 (41%)
	Total (% of 170)	52 (31%)	115 (69%)	167* (100%)

Note: *the difference in the number of dangerous solutions in Tables 2 and 4 is due to missing experts-patients pairs of usefulness-QoL impact estimates.

### The extent and type of solution diffusion

In our sample of respondents, we found that 32% (84) of all the patients and caregivers who reported solutions also reported investing efforts to share their solutions with others. After the initial screening, of the remaining 182 solutions (restricted sample), 55 (30%) respondents reported sharing their solutions with others (Table 5). We asked about seven types of diffusion effort that they might have undertaken. On average, they reported engaging in 1.5 of these diffusion activities in the restricted sample. 39 (71%) individuals reported only engaging in one of the seven possible diffusion activities. Diffusion effort was significantly higher (p < 0.05, two-tailed *t*-test) in the case of innovations that were new to the world than in the case of redevelopments that were only new to the individual patient/respondent, but only in the non-restricted sample. In the restricted sample there was no statistically significant difference between the two groups.

**Table 5:** **The patients’ solution sharing activities**

	**Non-restricted sample (n = 263)**		**Restricted sample (n = 182)**	
**Solution sharing activity**	**New to the world**	**New to the respondents**	**New to the world**	**New to the respondents**
	**(n = 19)**	**(n = 65)**	**(n = 19)**	**(n = 36)**
Shown it to other patients	89%	88%	89%	92%
Shown it to medical professionals	5%	6%	5%	2%
Shared the info on a website/blog/social network	37%	22%	37%	28%
Shared it through media	16%	5%	16%	8%
Shown it to commercial entities	11%	2%	10%	3%
Spent time and/or money to help others (people, companies) use the solution	11%	3%	10%	5%
Made a manual or documentation that helps using the solution	5%	3%	5%	5%

Considering the full-sample, the most common mode of sharing was patient-to-patient, reported by 74 individuals – 88% of those who shared a solution. Interestingly, only 5 individuals - 6% of the respondents who shared solutions – shared their solutions with their doctors. Sharing with commercial firms occurred in 3 cases. In four cases, patients or caregivers spent time or money to help diffuse their innovations. In three of these cases, the developers also reported making a manual or documentation to help others use their solutions.

### What influences patient innovation and solution sharing

We next assess factors that are associated with patients’ likelihood of developing and sharing their novel developments.

Two factors may help predicting patient’s likelihood to innovate (model 1, Table 6). The coefficient next to perception of limitation on life imposed by their disease is positive and statistically significant (p < 0.05): a one-point increase in perceived limitations imposed by the disease increases odds of patient innovating by a factor of 1.3 (95% CI: 1.0 - 1.7), everything else held constant (model 1). Also, having a university degree increases likelihood of patient innovation, with an increase of odds of patient innovating by a factor of 2 (95% CI: 1.0 – 3.0; p < 0.05) (model 1).

**Table 6:** **Logit models of likelihood of patient innovation and solution sharing**

	**(1)**	**(2)**	**(3)**
		**Non-restricted sample**	**Restricted sample**
	Patient innovation	Solution sharing	Solution sharing
Disease prevalence	0.15 (0.15)	−0.13 (0.16)	−0.11 (0.20)
Disease burden	0.27** (0.13)	0.12 (0.14)	0.09 (0.19)
Respondent’s Age	0.13 (0.09)	0.25** (0.1)	0.21** (0.11)
Respondent’s Age squared	−0.002* (0.0)	−0.002** (0.0)	−0.002* (0.001)
Disease Duration	0.02 (0.05)	−0.07* (0.04)	−0.13*** (0.05)
Disease Duration squared	−0.001 (0.0)	0.002* (0.0)	0.002*** (0.001)
Gender	−0.51 (0.42)	−0.25 (0.49)	−0.29 (0.55)
Academic Degree	0.68** (0.3)	0.33 (0.27)	0.37 (0.30)
Employment status	−0.05 (0.38)	−0.13 (0.36)	0.41 (0.44)
Marital status	0.66 (0.4)	−0.01 (0.36)	−0.25 (0.45)
Medical patient community membership	0.39 (0.43)	0.29 (0.44)	0.30 (0.51)
Improvement in overall quality of life (before-after solution use)		0.52*** (0.11)	0.55*** (0.15)
Constant	−7.26*** (2.2)	−7.58*** (2.4)	−7.53*** (2.54)
Observations	485	231	159
Chi2	28.8	39.1	27.3
McFaden’s pseudo R^2^	0.08	0.16	0.18
c-statistics	0.72	0.77	0.77

Raw coefficients shown; Robust standard errors in parentheses.

*** p < 0.01, ** p < 0.05, * p < 0.1.

In models 2 and 3 we explored factors significantly associated with innovation sharing. Model 2 included the full sample of 263 reported solutions, while model 3 included only 182 reported solutions that passed the initial screening. The strongest predictor of information sharing was the observed difference in the respondents’ overall quality of life before and after using a solution, in both models 2 and 3. A one-point increase in the perceived difference increased odds of sharing the solution by a factor of 1.7 (95% CI: 1.4 - 2.1), everything else held constant (p < 0.01) (model 2). The effect was robust to the change in the sample size in both the magnitude and the statistical significance. Also, we found an inverted U relationship between the age and the likelihood of sharing a solution - the effect of age on the predicted probability becomes negative after the age of 55 (p < 0.05) (model 2). The non-linear effect of age on the solution sharing did not maintain the statistical significance level in the non-restricted sample, although both the sign and the coefficient remained the same (model 3). Also, a major difference between models 2 and 3 is that duration of a disease has positive and non-linear relationship with the solution sharing in model 3, which was not the case in model 2. The instability of the results for the age and disease duration with respect to the solution sharing suggests that further exploration is needed to clarify these effects.

## Discussion

Our first-of-type study has found that 36% of our sample of rare disease patients and/or their non-professional caregivers have self-developed improvements to the management of their diseases and that use of these, significantly improved their quality of life. Our expert clinical evaluators determined that 40 (22%) of these 182 claimed improvements were new-to-the-world. The rest were already known to medicine, although not to the patient or caregivers who redeveloped them.

We suggest that our results have significant implications for all stakeholders in the health care delivery process related to rare diseases. An estimated 6% to 8% of the world’s population – hundreds of millions of people - are afflicted by rare diseases. It is known that commercial innovation efforts on these patients’ behalf are negatively affected by small market sizes. Our finding that 8% of rare disease patients and/or their non-professional caregivers have developed valuable, new to the world innovations to improve their own care suggests that a massive, non-commercial source of medical innovations exists. It is today largely hidden due to a lack of diffusion efforts by innovating patients, but efforts could be made to change this situation.

Significant positive relationships were found between the limitations caused by an illness, and also education level and likelihood of innovation. Both findings are in line with findings from other studies of innovation patterns among individual citizens [6,7,20]. We also found that the extent of improvement of patient quality of life from their developments is significantly positively associated with efforts to diffuse their innovations to others. Our data uncovered a potential principal-agent problem: patients systematically valuing different innovations than doctors do, as suggested by the observed differences between patient and clinician evaluations of usefulness (Table 4). In further work, we suggest that the sources of this difference should be explored. It is possible, we think, that clinicians assess the usefulness of innovations primarily in terms of whether they affect the clinical course of a disease in beneficial ways. In contrast patients may greatly value innovations that may have no impact on the course of their disease, but that improve their comfort or other aspects of their quality of life while living with their disease.

Recall that we found that most patient improvement development efforts were devoted to re-inventing known solutions of which they were not aware: only 22% of the solutions patients and caregivers developed were judged new to the world by our expert clinician raters. This suggests that information on known solutions, as well as novel ones, is today poorly diffused, and/or that information provided by clinicians is poorly absorbed by many patients. The duration of typical medical appointments are quite limited, and our finding may be due to this. A possibly related finding is that only 6% of patients reported describing their innovations to their clinicians.

Direct patient-to-patient information sharing was the most common mode of innovation diffusion and was reported by 88% of those who shared solutions. The second most common mode of sharing was via blogs/websites/social networks, reported by 25% of those who shared solutions. Rare disease patients and caregivers are known to actively use the Internet and social networks to find and provide help and connect with others patients and caregivers [21,22]. As 85% of the respondents in our sample use the Internet, and of these 71% use social networks, the reported level of solution sharing over the Internet appears low. Non-sharing of potentially useful solutions developed by individual citizens has been identified as a market failure in the user innovation literature [23]. Future research should explore diffusion incentives and effective channels for diffusion, taking into consideration that different age groups may have different requirements and behavioral patterns.

While our sample offers technically simple solutions, patient-developed solutions can also be technically very advanced. As illustration, consider the innovation (not found in this study sample) developed by a hydraulics engineer with Marfan syndrome. This individual had an aortic root aneurysm. Drawing upon his hydraulics engineering background, he developed a personalized external aortic support made from mesh to prevent a progressively enlarging aorta from prematurely ending his life [16,24]. More precisely, the patient assembled a medical and technical team and raised research and development money to further implement and diffuse his innovation. Upon development, his surgeon successfully installed the aortic support, and the clinical advantage was clear. To date, the novel treatment has been successfully applied to more than 40 patients in the period from 2004 to 2014 [25].

Given the novelty and medical value of some patient-developed innovations, as documented in this and other studies, we suggest that further research on this topic should be conducted. It would also be useful, we suggest, to develop and experiment with systems to collect and medically evaluate novel solutions developed by patients, to identify those worthy of further testing, improvement, and diffusion.

### Study limitations

This study into innovation development by patients and caregivers has several limitations. First, our sample of patients and caregivers are retrieved from a population of those who have already reached out towards the rare disease association for help via a helpline. These individuals may be more or less likely to innovate than those who did not make contact. Also, our respondents are dominantly women, indicating that female patients or caregivers are more likely to reach out for help through this particular patient association. This creates a modest conservative bias in our findings regarding innovation levels among patients and caregivers.

Second, telephone interviews rather than site visits are likely to understate the level of patient and caregiver innovations that actually exist. Individuals often do not recognize their activities as innovative, or do not report novel developments they make. This limitation makes the observed frequency of patient innovation a lower boundary for the observed population.

Third, self-assessment of satisfaction with a self-developed solution is known to be inflated by the pride individuals take in their self-developed solutions [26]. In addition, our quality of life measure is a single item scale that makes it open to individual interpretation and limits the ability to compare it across different individuals. This item does capture how people feel about their solutions, but caution is needed in interpreting the results.

## Conclusions

We found that 36% of our sample of rare disease patients and/or their non-professional caregivers had developed improvements to the management of their diseases that significantly improved their quality of life. Our expert clinical evaluators determined that 22% of these claimed improvements were new-to-the-world, and that the rest were already known to medicine, even if not to the patient or caregivers who redeveloped them. If further research finds that similar frequencies hold for the broader population, the estimated 6% to 8% of the world’s population afflicted by rare diseases may collectively offer a tremendous source of information on how to improve patient medical care. Their contributions may complement the efforts by policy makers, research entities, and producers, to help improve the difficult situation of rare disease patients, whose needs for innovations are often underserved.

It is important to continue investigating how rare disease patients and caregivers can be helped in their innovation activity, how innovations can be professionally assessed, and how patient-developed innovations determined to be of general value can be diffused for broader long-term public health benefits.

## Additional file

Additional file 1: Table S1. Data description.

## References

[1] Rodwell C, Aymé S, eds, “2014 Report on the State of the Art of Rare Disease Activities in Europe”, July 2014, European Union. (http://www.eucerd.eu/upload/file/Reports/2014ReportStateofArtRDActivities.pdf).

[2] Westermark K, Holm BB, Söderholm M, Llinares-Garcia J, Rivière F, Aarum S, Butlen-Ducuing F, Tsigkos S, Wilk-Kachlicka A, N’Diamoi C, Borvendég J, Lyons D, Sepodes B, Bloechl-Daum B, Lhoir A, Todorova M, Kkolos I, Kubáčková K, Bosch-Traberg H, Tillmann V, Saano V, Héron E, Elbers R, Siouti M, Eggenhofer J, Salmon P, Clementi M, Krieviņš D, Matulevičiene A, Metz H (2011). European regulation on orphan medicinal products: 10 years of experience and future perspectives. Nat Rev Drug Discov.

[3] Song P, Gao J, Inagaki Y, Kokudo N, Tang W (2012). Rare diseases, orphan drugs, and their regulation in Asia: current status and future perspectives. Intractable Rare Dis Res.

[4] Griggs RC, Batshaw M, Dunkle M, Gopal-Srivastava R, Kaye E, Krischer J, Nguyen T, Paulus K, Merkel PA (2009). Clinical research for rare disease: opportunities, challenges, and solutions. Mol Genet Metab.

[5] Acemoglu D, Linn J (2004). Market size in innovation: theory and evidence from the pharmaceutical industry. Q J Econ.

[6] von Hippel E, de Jong JPJ, Flowers S (2012). Comparing business and household sector innovation in consumer products: findings from a representative study in the United Kingdom. Manage Sci.

[7] De Jong JPJ, von Hippel E, Gault F, Kuusisto J, Raasch C. The diffusion of consumer-developed innovations: patterns in Finland. SSRN Electron J. 2014:1–30. http://papers.ssrn.com/sol3/papers.cfm?abstract_id=2426498.

[8] Ogawa S, Pongtanalert K (2011). Visualizing invisible innovation continent: evidence from global consumer innovation surveys.

[9] Oliveira P, von Hippel E (2011). Users as service innovators: the case of banking services. Res Policy.

[10] van der Boor P, Oliveira P, Veloso F (2014). Unusual suspects? Innovation by users in developing countries: evidence from mobile banking services. Res Policy.

[11] Dyson E (2009). Why participatory medicine?. J Particip Med.

[12] Ferguson T. E-patient: how they can help us heal healthcare. 2007

[13] Hibbard J, Stockard J, Mahoney ER, Tusler M (2004). Development of the Patient Activation Measure (PAM): conceptualizing and measuring activation in patients and consumers. Health Serv Res.

[14] Rozenblum R, Bates DW (2013). Patient-centred healthcare, social media and the internet: the perfect storm?. BMJ Qual Saf.

[15] Frydman G (2009). Patient-driven research: rich opportunities and real risks. J Particip Med.

[16] Habicht H, Oliveira P, Shcherbatiuk V (2012). User innovators: when patients set out to help themselves and end up helping many. Die Unternehmung.

[17] Oliveira P, Canhão H, Lakhani K, Harhoff D (2015). Users as service innovators: evidence from banking to healthcare. Revolutionizing innovation.

[18] Kuenne C, Akenroye T, Moeslein K (2013). Online innovation intermediaries in healthcare. European Conference on Information Systems (ECIS) 2013 Proceedings.

[19] ISO (2011). Assistive products for persons with disability — classification and terminology.

[20] Lüthje C, Herstatt C, von Hippel E (2005). User-innovators and “local” information: the case of mountain biking. Res Policy.

[21] Fox S (2011). Peer-to-peer healthcare.

[22] White W, Calhoun C, Holovach J, Marie J, Buchanan L, Sames L et al. Uncommon Challenges; Shared Journeys. Siren Press; 2011.

[23] von Hippel E, DeMonaco HJ, de Jong JPJ. Market failure in the diffusion of user innovations: the case of “Off-Label” innovations by medical clinicians. SSRN Electron J 2014:1–33.

[24] Treasure T, Pepper J, Golesworthy T, Mohiaddin R, Anderson RH (2012). External aortic root support: NICE guidance. Heart.

[25] Treasure T, Pepper J (2015). Personalised External Aortic Root Support (PEARS) compared with alternatives for people with life-threatening genetically determined aneurysms of the aortic root. Diseases.

[26] Franke N, Schreier M, Kaiser U (2010). The “I Designed It Myself” effect in mass customization. Manage Sci.

