# Supplementary material for: Innovation by patients with rare diseases and chronic needs
Source: Orphanet J Rare Dis. 2015 Apr 9;10:41. doi: 10.1186/s13023-015-0257-2 (PMC4404234; doi:10.1186/s13023-015-0257-2)
Supplement: Additional file 1: Table S1. — Data description. [file 13023_2015_257_MOESM1_ESM.docx]

Table S1 Data description

| *Nr.* | | *Variable* | | *Type* | *Source* | |  |
| --- | --- | --- | --- | --- | --- | --- | --- |
|  | Reported innovation | | Binary | | Survey question: Did you ever develop new or modify an existing medical device, treatment, therapy, or something in your behaviour to help you cope with your disease? (0-no; 1-yes); Additional questions were asked to identify the solution. | | |
|  | Patient innovator | | Binary | | Did the respondent propose solution that has been evaluated as novel by external evaluators? (0-no; 1-yes) |  |  |
|  | Information sharing | | Binary | | Survey question: Did you do anything to share your solution with others? (0-no; 1-yes) |  |  |
|  | Innovation sharing efforts | | Ordinal | | Sum of 7 dichotomous variables, each representing a sharing activity: 1.sharing with patients, 2. with medics, 3. with companies, 4. with traditional media, 5. publishing an online post, 6. creating a manual or a document, 7. investing time or money to show others how to use it. |  |  |
|  | Disease prevalence | | Ordinal | | The disease’s prevalence (collected from publicly available databases*); inversely coded - the more rare is the disease (order of magnitude), the higher the value: 1 to 5; >1 / 1000; 1-9 / 10 000; -9 / 100 000; 1-9 / 1 000 000; <1 / 1 000 000); for 5 of the diseases in our sample the prevalence was not found; |  |  |
|  | Disease burden | | Ordinal | | Survey question: To what extent does the disease impose limitations to your daily life (1- not at all; to 5 – extremely; Likert scale) (independently of your solution? |  |  |
|  | Years with the disease | | Continuous | | Survey questions: How long have you been diagnosed with the disease? |  |  |
|  | Squared years with the disease | | Continuous | | Squared disease duration (variable 7) |  |  |
|  | Age (respondent’s and patient’s) | | Continuous | | Survey question: How old are you? How old is the patient?  Only the respondent’s age was kept in the analysis – of the innovator. |  |  |
|  | Squared Age | | Continuous | | Squared respondent’s age |  |  |
|  | Gender(respondent’s and patient’s) | | Binary | | Survey questions: Your gender? The patient’s gender? (0-male; 1-female) |  |  |
|  | Quality of Life before the innovation | | Ordinal | | Survey question: What was your overall quality of life before you developed your solution (1- extremely bad; to 7– extremely good; Likert scale)? |  |  |
|  | Quality of Life after the innovation | | Ordinal | | Survey question: What was your overall quality of life after you started using the solution that you developed (1- extremely bad; to 7– extremely good; Likert scale)? |  |  |
|  | The respondent’s before-after difference in Quality of Life | | Ordinal | | This variable is obtained by subtracting the variable “Quality of Life before the innovation” from the “Quality of Life after the innovation”. |  |  |
|  | University Degree | | Ordinal | | Survey questions: Your highest education level / academic degree? (0-less than university; 1-Bachelor or Graduate degree) |  |  |
|  | Employment status | | Binary | | Survey question: Your professional status: Employed; Student; Unemployed; Retired; Stay-home; other; (0-not active employment; 1-employed) |  |  |
|  | Marital status | | Binary | | Survey question: Your marital status? married; single; divorced; de facto union marriage; widow(er); (0-single; 1-married or with a partner) |  |  |
|  | Community membership | | Binary | | Survey questions: Are you a member of any health related association? (0-no; 1-yes) |  |  |

*Note: Sources for the Rareness variable: online search of medical databases, books and journals (e.g. Medline, National Institute of Health, Orphanet, Eurordis, BMJ, etc.)
